# Hepatic Arteriography and C-Arm CT-Guided Ablation (HepACAGA) to Improve Tumor Visualization, Navigation and Margin Confirmation in Percutaneous Liver Tumor Ablation

**DOI:** 10.1007/s00270-023-03545-4

**Published:** 2023-09-13

**Authors:** Maarten L. J. Smits, Rutger C. G. Bruijnen, Philip Tetteroo, Evert-jan P. A. Vonken, Martijn R. Meijerink, Jeroen Hagendoorn, Joep de Bruijne, Warner Prevoo

**Affiliations:** 1 Department of Radiology and Nuclear Medicine, University Medical Center Utrecht, Utrecht, The Netherlands; 2 Department of Radiology, Amsterdam University Medical Center, Amsterdam, The Netherlands; 3 Department of Surgery, University Medical Center Utrecht, Utrecht, The Netherlands; 4 Department of Gastroenterology and Hepatology, University Medical Center Utrecht, Utrecht, The Netherlands

**Keywords:** Liver, Ablation, CTHA, Catheter, Hepatocellular carcinoma, Colorectal cancer, Microwave, Cone beam CT

## Abstract

**Purpose:**

We present a technique that combines Hepatic Arteriography with C-arm CT-Guided Ablation (HepACAGA) to improve tumor visualization, navigation and margin confirmation for percutaneous ablation of liver tumors.

**Materials and Methods:**

All consecutive patients scheduled for HepACAGA between April 20th, 2021, and November 2nd, 2021, were included in this retrospective, cohort study. HepACAGA was performed in an angiography-suite under general anesthesia. The hepatic artery was catheterized for selective contrast injection. C-arm CT and guidance software were then used to visualize the tumor and the microwave antenna was inserted during apnea. Pre- and post-ablation C-arm CTs were performed and ablation margins assessed. Technical success, antenna placement deviation, number of repositions, tumor recurrence, and safety were evaluated. Technical success was defined as a tumor that was ablated according to the HepACAGA technique.

**Results:**

A total of 21 patients (28 tumors) were included. The main tumor type was colorectal cancer liver metastases (11/21, 52%), followed by hepatocellular carcinoma (7/21, 33%), neuroendocrine tumor metastases (1/21, 5%), and other tumor types (2/21, 10%). The technical success rate was 93% (26/28 tumors) with two small hypovascular lesions unable to be identified. A single microwave antenna was used in all patients. The median antenna placement deviation was 1 mm (range 0–6 mm). At a median follow-up time of 16 months (range 5–22 months), there was no tumor recurrence in any patient. Safety analysis showed a complication rate of 5% grade 2 and 5% grade 3.

**Conclusion:**

HepACAGA was demonstrated to be a safe and effective percutaneous ablation technique, without any local tumor recurrence in this study.

**Graphic Abstract:**

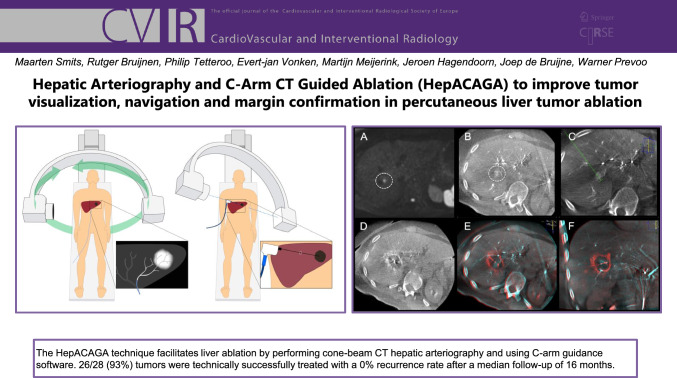

## Introduction

Percutaneous image-guided thermal ablation is a valuable treatment option for a variety of tumors in the liver, mainly hepatocellular carcinoma (HCC) and liver metastases from colorectal carcinoma (CRC) [1, 2].

Ultrasound and CT are the primary modalities used for needle-placement guidance during thermal ablation procedures. CT is advantageous in cases where tumors undectectable on ultrasound due to factors such as cirrhosis, obesity, or a suboptimal location (e.g., obscured by the lung or intestines). Nevertheless, CT has limitations; not all tumors are visible, and even when they are visible on contrast-enhanced CT, the contrast agent may have washed out prior to antenna insertion. Safety considerations also limit the number of contrast-enhanced series that may be performed. Consequently, puncturing liver tumors often entails a ‘semi-blind’ approach, navigating on contours of the continuously moving liver.

An important milestone in this therapy was the introduction of transcatheter hepatic arteriography (CTHA) to facilitate CT-guided puncture [3, 4]. This technique involves repeated administration of a small amount of iodine contrast agent (typically 15–30 ml) into the hepatic artery throughout the procedure. This approach notably enhances the differentiation between tumors and surrounding tissue and performs considerably better than intravenous injection of contrast. Moreover, it is possible to perform numerous contrast-series before the maximum dose of contrast medium is reached.

An inherent drawback of this catheter-ablation method is that it is a two-step process; initial catheter placement is performed under X-ray guidance in the angiography suite and thereafter the ablation is performed in the CT room. Facilities without a hybrid angiography-CT room must therefore transport patients between these rooms, which is disadvantageous due to the risk of catheter displacement, contamination of the sterile field and logistical challenges. (Both rooms require reservation.)

In this paper, a modified technique is presented: Hepatic Arteriography and C-arm CT-Guided Ablation (HepACAGA) using commonly available navigation and CT-fusion software to perform both the placement of the hepatic artery catheter and the ablation itself in the angiography suite.

## Materials and Methods

### Ethical Approval

This study was approved by the local ethical institutional review board, and the need for informed consent was waived.

### Patients

All consecutive patients scheduled for liver tumor ablation between April 20th, 2021, and November 2nd, 2021, were included in this retrospective cohort study. All patients were discussed in a multidisciplinary tumor board before being referred for thermal ablation.

### Treatment

#### Procedure Preparation

**Fig. 1:**
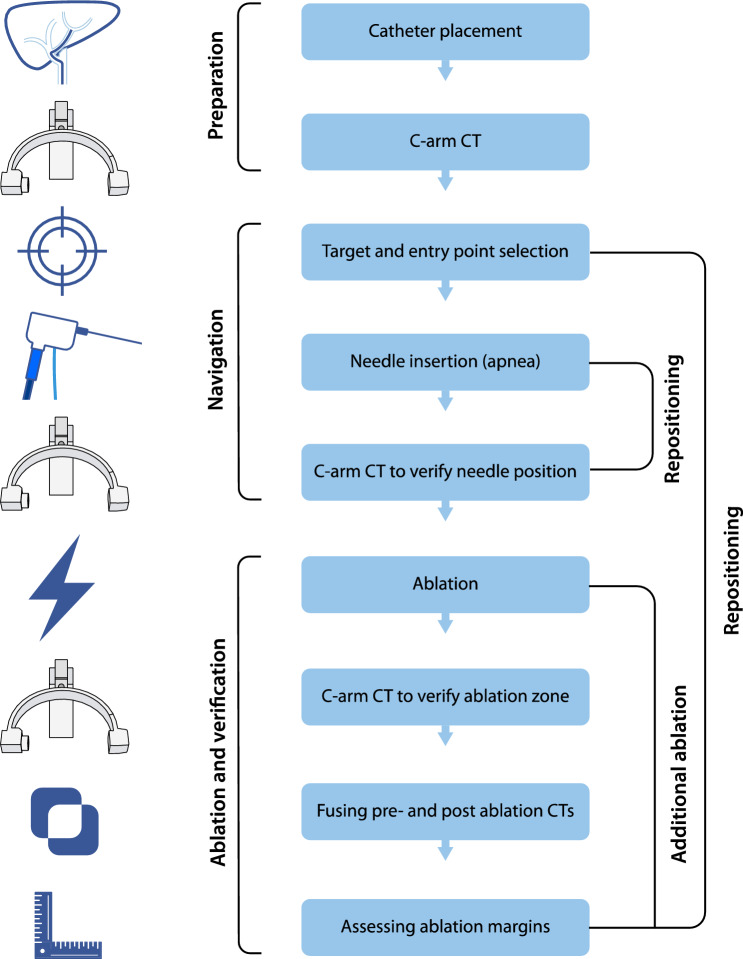
Stepwise schematic of how the HepACAGA technique is performed

**Fig. 2:**
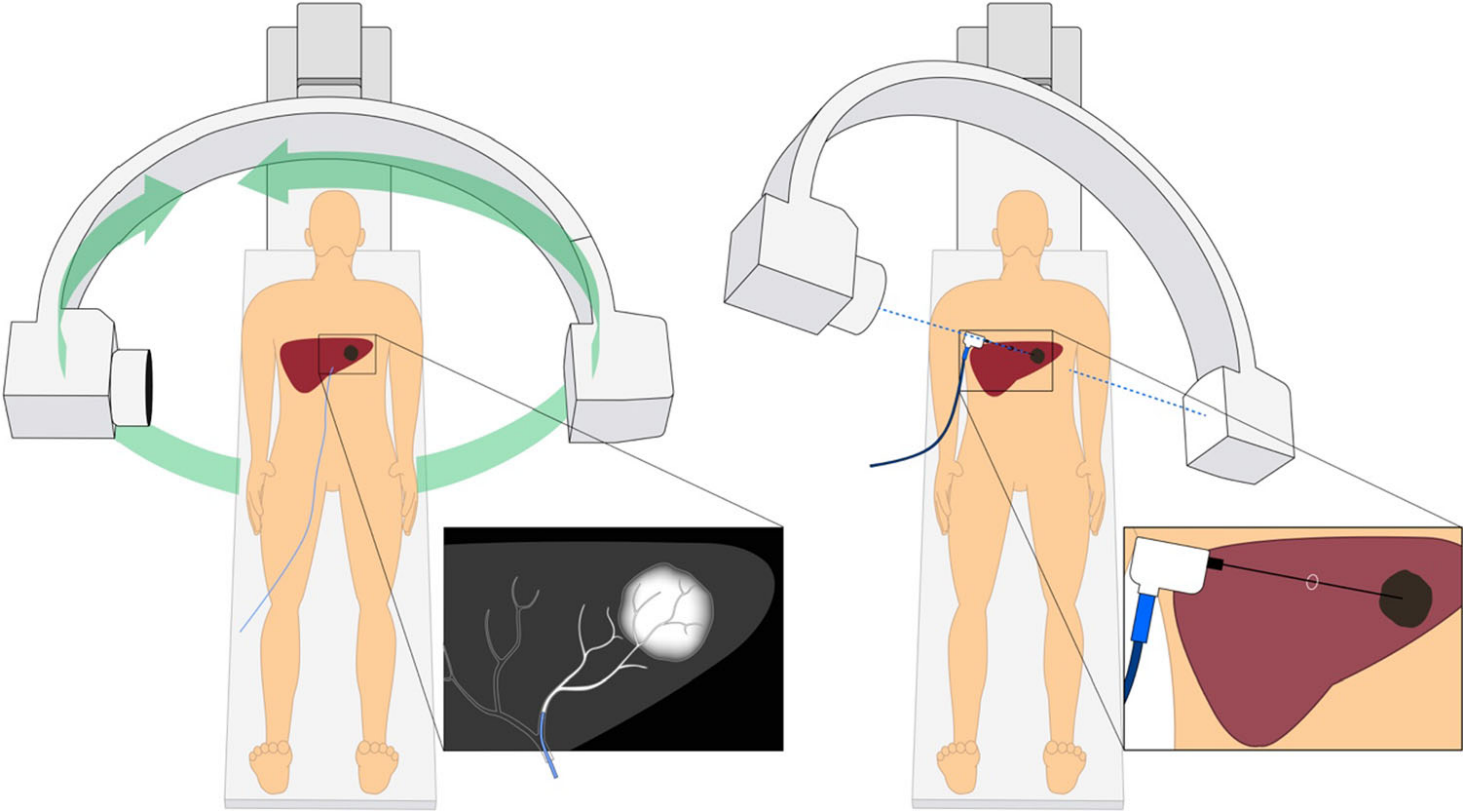
Schematic of the HepACAGA technique. Left panel: C-arm CT is obtained during contrast injection through a catheter placed in the hepatic artery. Left insert: tumor enhancement due to selective contrast injection. Right panel: the C-arm is automatically positioned in a way that the entry point and target are aligned (entry point view). The entry point and target are projected on the live fluoroscopy images. Right insert: positioning of the microwave antenna through the projected entry point into the target

The steps of the HepACAGA technique are illustrated in Figs. 1 and 2. Patients were admitted on the day of ablation. Treatment was performed in an angiography suite under general anesthesia. General anesthesia was required in order to obtain apnea during the procedure. Patients were positioned supine, with an arms-down posture. Care was taken to position tubing and other lines in such a way that the C-arm could rotate around the patient unobstructed.

#### Transarterial CT Arteriography

The common femoral artery was punctured and a catheter was advanced into the common, proper, left or right hepatic artery, depending on the tumor location. C-arm CT was performed with simultaneous intra-arterial contrast injection through the hepatic catheter (hepatic arteriography). The duration of the C-arm rotation was 10 s. Contrast agent (Visipaque 320 mg/ml, 2:1 diluted with NaCl) was injected using a pump filled at 1.0–2.0 ml/s with 10 s delay. The total amount of contrast agent used varied between 13–27 ml. Where tumors were not visible in the arterial phase, a late-phase C-arm CT was performed with 40 s scanning delay.

#### Planning and Navigation

**Fig. 3:**
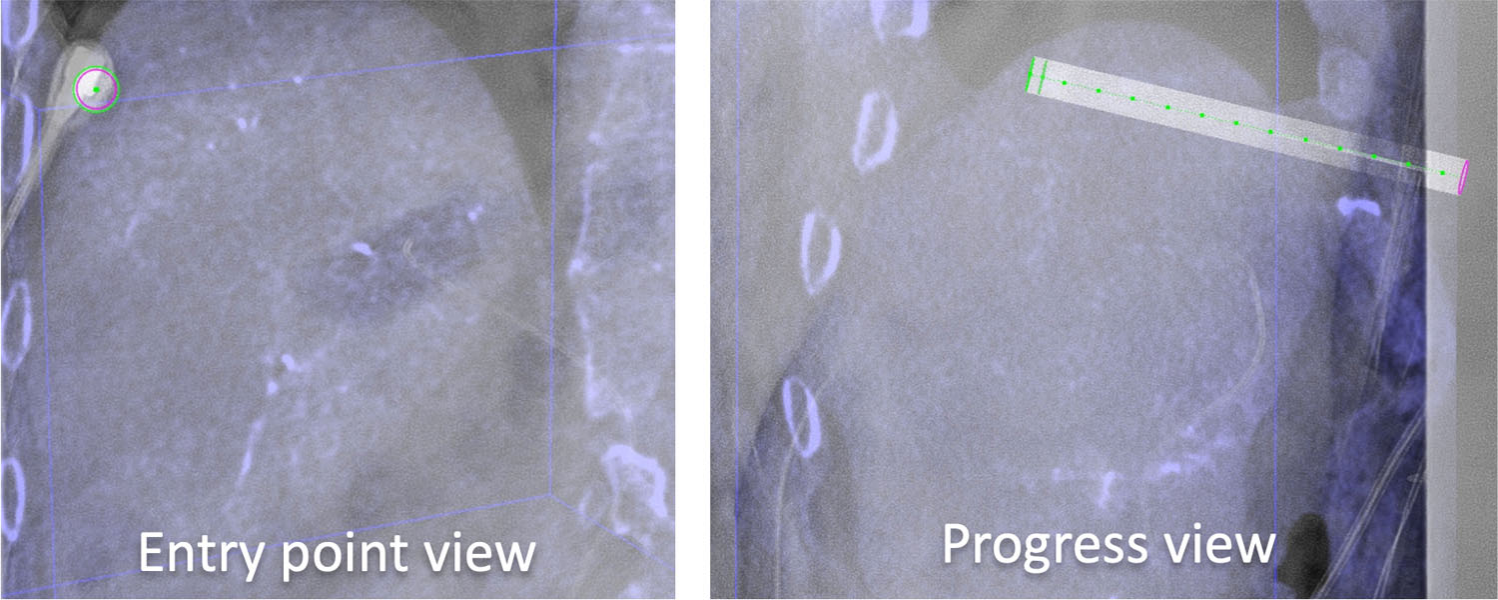
navigation with XperGuide software (Philips). Left panel: entry point view in which the entry point (pink circle) and target (green circle) are projected on top of each other. The microwave antenna is now held directly in line with the projected needle path. Right panel: progress view showing the needle path at a 90 degree angle allowing for assessing needle depth

C-arm CT was checked for visualization of the target tumor, the presence of any new tumors, and the position of non-target structures (e.g., ribs, bowel, stomach, large veins, bile ducts). The needle trajectory was planned with C-arm navigation software (XperGuide, Philips, Best, The Netherlands) (see Fig. 3). Subsequently, the antenna was advanced in a straight line from the entry point to the target, under real-time fluoroscopy-guidance. Placement of the antenna was performed in apnea in order to mimic the position of the liver during planning-C-arm CT. Apnea was obtained by pausing the ventilator, allowing the lungs to deflate. After antenna placement, C-arm CT with hepatic arteriography was repeated to confirm correct placement. If the antenna had deviated from the planned trajectory, the antenna was repositioned.

#### Microwave Ablation

The Emprint® HP (Medtronic, Dublin, Ireland) microwave generator and antennas were used. The desired ablation size was determined by measuring the largest distance from the antenna to the edge of the tumor perpendicular to the antenna in two planes, and subsequently by measuring the length of the tumor along the direction of the antenna. A margin of 5–10 mm was added on all sides if possible, resulting in a desired ablation size.

#### Verification and End of Procedure

**Fig. 4:**
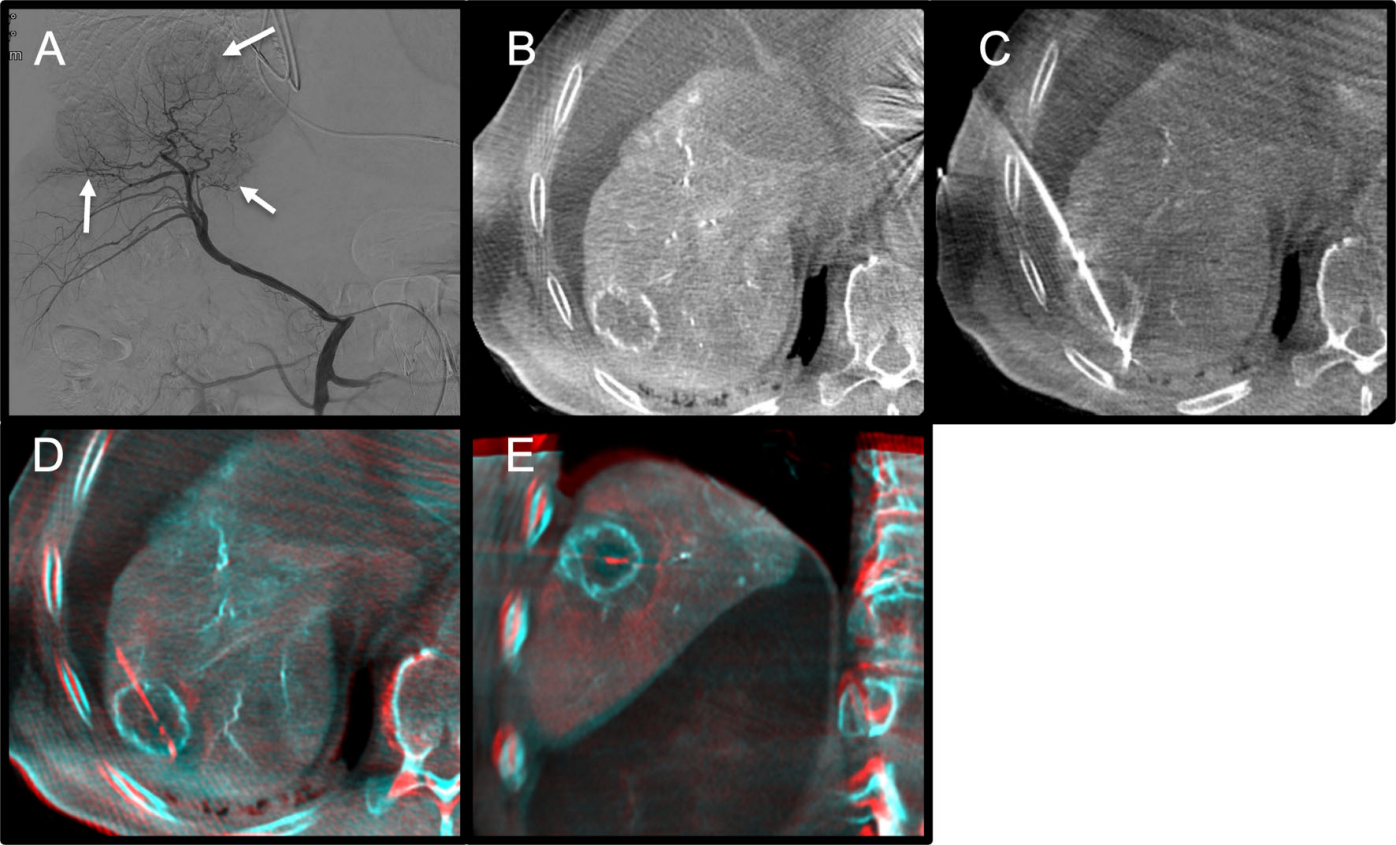
Example of a hypervascular hepatocellular carcinoma (HCC) ablation. **A**. Digital subtraction angiography of the right hepatic artery shows three hypervascular HCCs. **B**. C-arm CT with hepatic arteriography shows the first target lesion. **C**. Antenna in position. **D** and **E**. Fused pre- and post-ablation C-arm CTs depicting ablation margins

**Fig. 5:**
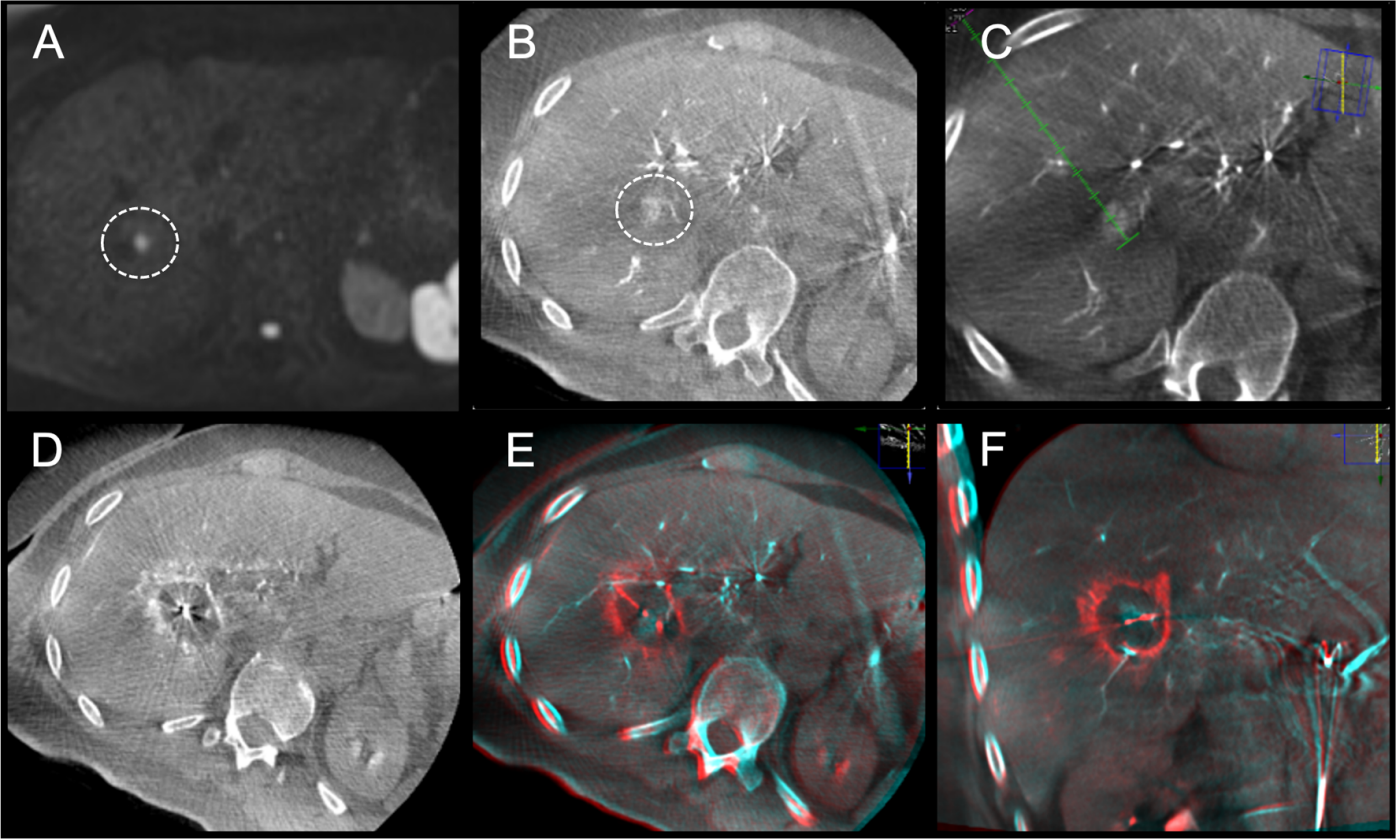
Example of a colorectal liver metastasis ablation. **A**. Solitary colorectal cancer liver metastasis only visible on diffusion weighted MRI (not visible on contrast-enhanced MRI). **B**. C-arm CT with hepatic arteriography clearly depicts the 11 mm lesion. **C**. Planning of trajectory. D. Ablation zone depicted with C-arm CT hepatic arteriography directly after ablation for 8 min at 120W. E and F. Fused pre- and post-ablation C-arm CTs in axial and coronal view show adequate margins

C-arm CT with hepatic arteriography was performed directly following the ablation to assess the ablation zone. Pre- and post-ablation C-arm CTs were fused using the XperGuide software to verify adequate margins (see Figs. 4 and 5). If margins were inadequate, the antenna was repositioned and additional ablation was performed. After ablation, the antenna was retracted during tract ablation. The catheter and sheath were removed, and the arterial puncture site was closed.

### Outcome Assessment

The consensus guidelines for end-points in image-guided tumor ablation were followed for reported outcomes [5]. The main outcome of this study was technical success rate of performing the HepACAGA technique. Secondary outcomes were antenna placement deviation, number of repositions, time of procedure, tumor recurrence, and safety.

#### Technical Success and Procedure

**Fig. 6:**
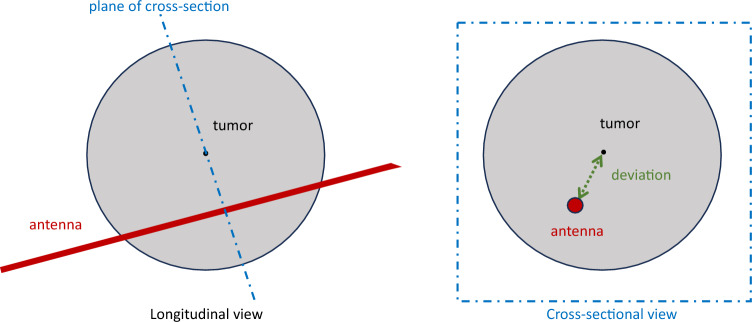
Method of measuring antenna placement deviation. Left panel: multi-planar reconstruction along the antenna (longitudinal view). Right panel: reconstruction tangential on the axis of the antenna to measure the deviation between antenna and tumor center (green dotted line)

**Fig. 7:**
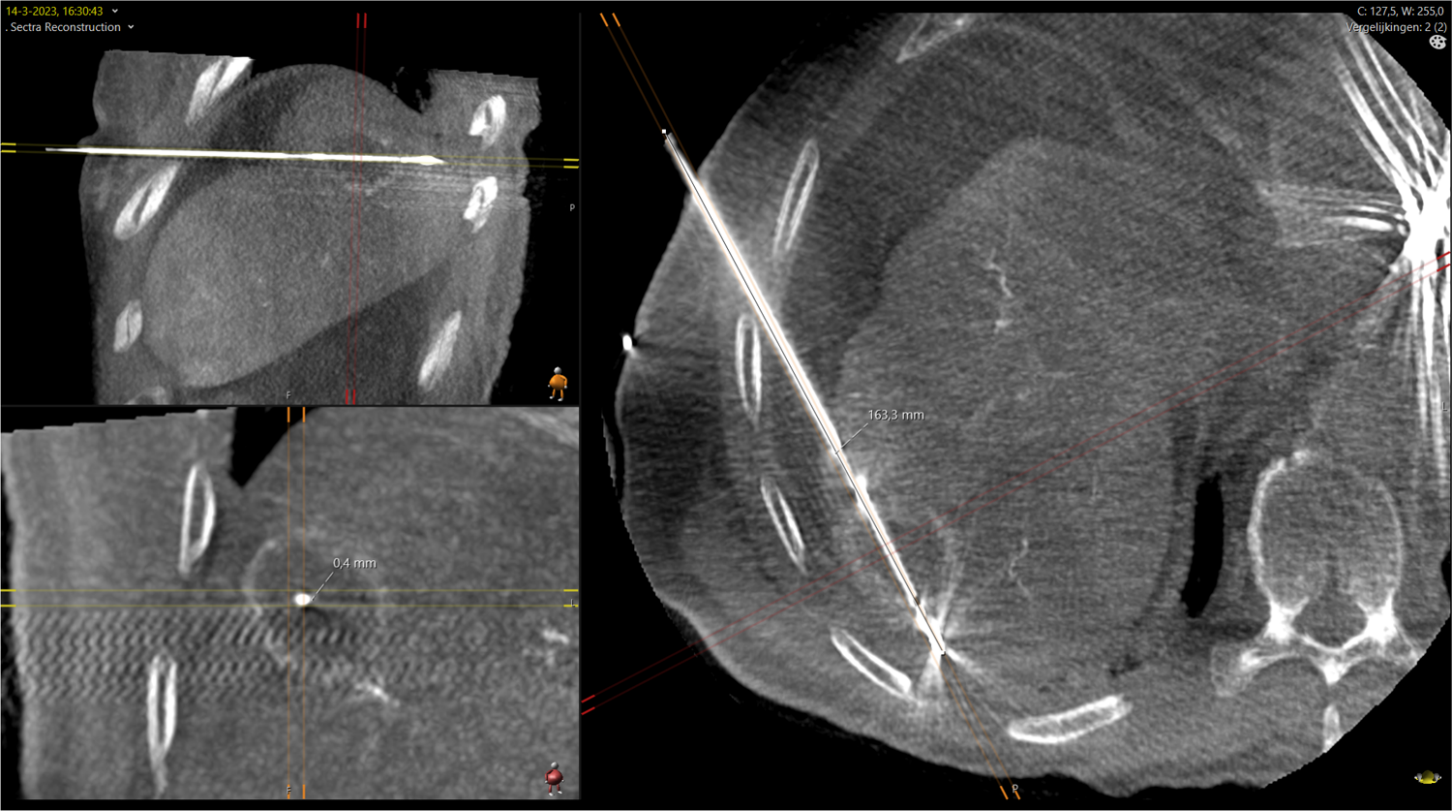
Method of measuring antenna deviation (same patient as Fig. 4). Multiplanar reconstruction in three orthogonal planes aligned with the antenna trajectory. The lesion is visible as an rim-enhancing mass. The antenna placement error was measured as the distance between the antenna and the center of the tumor on the short axis of the antenna trajectory (left lower panel). In this case the error was 0 mm at an antenna insertion depth of 163 mm

Technical success was defined as a tumor that was ablated according to the HepACAGA technique. C-arm CT after placement of the antenna (but before ablation) was used to estimate the antenna placement error. To achieve this, the distance of the antenna to the center of the tumor was measured using multiplanar reconstructions (see Figs. 6 and 7). Measurements were performed independently by two non-blinded interventional radiologists (Maarten Smits and Warner Prevoo). The average of both readers’ error-measurements were used. The antenna insertion depth was measured on C-arm CT. Number of repositions were derived from the number of C-arm CTs performed (repositioning requires a new C-arm CT). Several timing parameters were recorded (patient arrival, start of anesthesia, start of procedure, end of procedure, end of anesthesia, departure of patient). In-room time was defined as the interval between patient arrival and patient departure. Procedure time was defined as the interval between the start of the procedure (groin puncture) and the end of the procedure (placing the vascular closure device).

#### Tumor Recurrence

CT, MRI or ^18^F-FDG PET-CT was used to evaluate treatment effect and possible tumor recurrence during follow-up. Tumor recurrence was derived from the radiology reports (in order to prevent potentially biased reading by investigators). Any sign of residual or recurring tumor at the site of ablation was considered tumor recurrence.

#### Safety

Patient records were screened for complications up to 30 days post-treatment. Complications were graded according to CTCAE v5 [6].

#### Statistics

Data were summarized using descriptive statistics. Due to the small number of patients no detailed statistical analyses were performed.

## Results

### Patient Inclusion

A total of 21 patients were included and 28 tumors were ablated. The median age of the included patients was 67 years (range 16–87) with a median BMI of 26 (range 16–36). The main tumor type was CRC (11/21, 52%), followed by HCC (7/21, 33%), neuroendocrine tumor metastases (1/21, 5%), and other tumor types (2/21, 10%). Additional demographic data are listed in Table 1.

**Table 1:** Demographics

Demographics	n	%
Patients	21	–
Tumors	28	–
*Sex*		
Male	15	71
Female	6	29
Median age (years, range)	67	16–87
Median BMI (kg/m^2^, range)	26	16–36
*ASA*		
1	0	0
2	11	52
3	10	48
*Tumor type*		
CRC metastases	11	52
HCC	7	33
NET metastases	1	5
Breast cancer metastases	1	5
Cholangiocarcinoma metastases	1	5

### Technical success

The HepACAGA technique was successfully performed in 26/28 tumors (93%) and in 19/21 patients (90%) (Table 2). In the remaining two procedures the lesions could not be clearly identified on C-arm CT and the ablations were performed under ultrasound guidance.

**Table 2:** Procedural characteristics

Procedural characteristics	n	%
Procedures	21	–
Technically successful HepACAGA procedures	19	90
Unsuccessful HepACAGA procedures	2	10
Number of treated tumors	26	–
Mean tumor size (mm, range)	18	5–37
Mean insertion depth (mm, range)	111	59–163
Median power (Watt, range)	100	80–150
Median ablation duration (minutes, range)	6	3–15
Mean procedure duration (minutes, range)		
Start till finish of procedure per tumor	73	30–106
Start till finish of procedure overall	90	51–170
Total time in the angio suite	137	93—236
Repositions		
0	21	81
1	5	19
Antenna placement deviation (median, range)	1	0–6
Radiologist 1	1	0–4
Radiologist 2	1	0–6
Deviation not evaluable (no. tumors)	7	27

### Procedure

All procedures were performed in entirety in the angiography suite. There were no catheter dislocations in any of the procedures. The median tumor size was 18 mm (range 5–37), and the median antenna insertion depth was 111 mm deep (range 59−163 mm). After insertion and optional repositioning, the median antenna placement error was 1 mm (range 0–5 mm). The antenna required no repositioning in 21/26 (81%) tumors and a single repositioning in 5/26 (19%) tumors. The median ablation time was 6 min (range 3–15 min) at a median power of 100 W (range 80–150 W). The mean procedure time per ablated tumor was 73 min (range 30–106 min). The mean overall procedure time was 90 min (range 51–170 min), and the mean in-room time was 137 min (range 93–236 min). Median dose area product for the entire procedure was 137 Gy cm^2^ (range 58–333 Gy cm^2^).

### Tumor Recurrence

Median imaging follow-up was 16 months (range 5–22 months) (Table 3). Follow-up was performed with MRI in 11/19 patients and 8/19 patients received a combination of MRI, CT or ^18F^FDG-PET. No patients had signs of residual tumor or recurrent tumor at the site of ablation during follow-up (primary and secondary tumor recurrence rate 0%). Newly identified liver tumors falling outside the regions that were ablated were observed in 6/19 (32%) patients during follow-up. Of these six patients, two were eligible for re-treatment and were tumor-free following their second ablation. Additionally, three developed new or progressive extrahepatic tumors.

**Table 3:** Follow-up

Follow-up		
Median follow-up (months, range)	16	5–22
Follow-up modality		
MRI only	11	57%
MRI + CT	7	37%
MRI + ^18F^FDG-PET	1	5%
Patients with tumor recurrence (concerning the ablated tumor)	0	0%
Patients with new liver tumors (other than ablated tumors)	6	32%
Patients with progressive or new tumors outside the liver	3	16%
Complications		
Complicated procedures	2	10%
Uncomplicated procedures	17	90%
Grade 2	1	5%
Grade 3	1	5%

### Complications

Two complications were reported during follow-up that were potentially or probably related to the ablation. One patient developed a liver abscess at the ablation site requiring drainage, i.v. antibiotics and a prolonged hospital stay of 20 days after ablation. This patient had a biliodigestive anastomosis after a Whipple operation. Another patient exhibited pleural effusion and partial atelectasis of the right lung at 1-month follow-up scan, not requiring intervention. These findings were likely related to the ablation of a lesion in the liver dome. Both patients recovered fully. No procedure related deaths occurred.

## Discussion

This study reports on a novel technique for performing percutaneous liver tumor ablations; HepACAGA, which achieves a robust 93% successful ablation rate and a 0% tumor recurrence rate.

The concept of performing hepatic arteriography to facilitate liver tumor ablations is not new [3, 4, 7]. However, where previous studies have evaluated a two-step process involving transport of the patient from the angiography suite to CT for catheterization and ablation, respectively, HepACAGA expedites the treatment workflow by amalgamating both steps into one procedure that may be performed entirely in the angiography suite. This relies on navigation software to guide the puncture and semi-automatic registration software to assess tumor margins. These aspects of the technique are described in further detail below.

Firstly, since the procedure is performed entirely in the angiography suite, there is no need to transport the patient minimizing the risk of catheter luxation and removing the need to block a CT room. Also, any bleeding occurring during the procedure can be directly solved with embolization. Secondly, puncturing with C-arm guidance has the benefit of freedom in (multiplanar) angulation. It is easy to choose a puncture route out of the axial plane, for instance, with a caudo-cranial angulation, and even lesions high in the liver dome can be reached without traversing the lung or other non-target structures. This C-arm guidance is already installed on many angiography systems, obviating the need for expensive and often complex robotic systems [8–10]. A downside of this technique is that it requires fluoroscopy while the operator is placing the antenna, resulting in radiation exposure. Thirdly, HepACAGA allows for direct assessment of ablation margins. The ablation zone can be clearly depicted when performing a C-arm CT with hepatic arteriography directly after ablation. The ablation zone is then visible as an avascular area surrounded by a hyperemic rim. The XperGuide software is capable of semi-automatically fusing pre- and post-ablation C-arm CTs (see Figs. 4and5). Margins can be visually assessed and manually measured in multiple directions.

Overall, the enhanced visibility and advanced navigation options afforded by the method have expanded the scope of treatable lesions and allowed for treatment of lesions previously considered non-ablatable. Notably, deeply seated lesions or small lesions detectable only as diffusion restriction foci on MRI (and invisible on other MRI sequences, CT or ultrasound) can now be visualized and ablated with this technique.

A general downside of the hepatic angiography for ablation is that it requires vascular catheterization, which can prolong the procedure and comes with a small risk of puncture site bleeding. In this study, there were no complications related to catheterization. Even though catheterization initially adds time to the procedure, the total procedure time may be shorter than without the use of a catheter.

A downside of the HepACAGA technique is that it requires apnea when performing the C-arm CTs and when inserting the antenna. Several investigators have stated that complete tube disconnection or jet ventilation is required to get the liver into the same position for stereotactic puncture [11, 12]. In our practice, all patients were treated under general anesthesia and apnea was obtained by stopping the ventilator and allowing the lungs to deflate during at least 10 s delay before C-arm CT.

There are several ways in which HepACAGA can be further refined. In order to perform C-arm CT, the C-arm needs to be able to make an unobstructed orbit around the patient, which is sometimes impossible in obese patients. Considerable effort is therefore spent in patient positioning, removing arm supports and leading tubing, ECG leads, and i.v. lines along the patient’s left flank.

Image quality is also a concern with C-arm CT, as the images are often noisy and prone to artifacts [13]. While this was usually not an issue for the HepACAGA technique due to the high contrast between the target lesions and the surrounding tissue, improved CT-quality may aid in the detection and treatment of smaller or poorly enhancing lesions. A hybrid angio-CT set-up could greatly improve image quality. Yu et al. have demonstrated the feasibility of liver tumor ablation using CTHA in an angio-CT set-up [14]. This approach could potentially benefit from using C-arm fluoroscopy for guiding the puncture, as well as fusion software to assess ablation margins [15].

While primarily effective for hypervascular tumors, this technique also demonstrated success in hypovascular tumors. In practice, most tumors that appeared hypovascular on CT or MRI showed arterial hyperenhancement on hepatic arteriography C-arm CT. However, within this cohort, two small hypovascular colorectal metastases could not be clearly identified on hepatic arteriography C-arm CT. Therefore, an optional delayed phase C-arm protocol (40 s delay) was added that enabled the identification of tumors without any arterially enhancing component.

This study has several limitations. It is a retrospective study introducing a technique in a limited number of patients with heterogeneous tumor types. There was no control group, and the follow-up period was limited. Further studies are required to investigate the outcomes of this technique compared to existing techniques.

In conclusion, we present a technique to percutaneously treat liver tumors with Hepatic Arteriography and C-arm CT Guided Ablation (HepACAGA). In this cohort, the technique was demonstrated to be technically feasible, safe, and prevented local recurrence.
